# 

**Published:** 1880-10

**Authors:** 


					﻿Vol. 1. OCTOBER, 1880. No. 1O.
THE INDEPENDENT
JJraditmier:
ft 0 μ T H L Y J 0 U F( μ A L ,
DEVOTED TO
MEDICAL, SURGICAL, OBSTETRICAL,
DENTAL and HYGIENICAL
SCIENCE.
EDITED BY
HARVEY L. BYRD, A. M., M. D.,
AND
BASIL M. WILKERSON, D. D. S„ M. D.
“Alterco poscit opem res, et conjiirat amice”
BALTIMORE:
THE PRACTITIONER PUBLISHING CO.,
B. M. WILKERSON, Proprietor,
No. 68 North Charles Street.
4>2.OO Per Annum in Advance. - - Single Copies, 23 Cents.
ENTERED AT THE POSTOFFICE AT BALTIMORE, MD., AS SECOND-CLASS MATTER.
				

